# Anticancer Activity of Ethyl Acetate/Water Fraction from *Tanacetum vulgare* L. Leaf and Flower Extract

**DOI:** 10.3390/cimb47040215

**Published:** 2025-03-21

**Authors:** Inna Sulikovska, Ani Georgieva, Vera Djeliova, Katerina Todorova, Anelia Vasileva, Ivaylo Ivanov, Mashenka Dimitrova

**Affiliations:** 1 Department of Pathology, Institute of Experimental Morphology, Pathology and Anthropology with Museum, Bulgarian Academy of Sciences, Acad. G. Bonchev Str., 25, 1113 Sofia, Bulgaria; katerinagencheva@yahoo.com (K.T.); mashadim@abv.bg (M.D.); 2 Department of Molecular Biology of Cell Cycle, Institute of Molecular Biology “Acad. R. Tsanev”, Bulgarian Academy of Sciences, Acad. G. Bonchev Str., 21, 1113 Sofia, Bulgaria; vera@bio21.bas.bg; 3 Department of Bioorganic Chemistry and Biochemistry, Medical University of Sofia, Zdrave Str., 2, 1431 Sofia, Bulgaria; vasileva_anelia@abv.bg (A.V.); iivanov@medfac.mu-sofia.bg (I.I.)

**Keywords:** *Tanacetum vulgare*, ethyl acetate soluble fraction, HT-29 cells, flow cytometry, comet assay, Ehrlich’s carcinoma

## Abstract

The present paper aims to assess the antitumor activity of ethyl acetate/water soluble fraction obtained from the extract of *Tanacetum vulgare* L. leaves and flowers (EATV). The chemical composition of EATV was determined by LC-HPMS. In vitro studies were performed on the HT-29 cell line (human colorectal carcinoma). The effect on the cell cycle and the pro-apoptotic activity were evaluated using flow cytometry analyses. Genotoxicity was analyzed by the comet assay. In vivo antitumor potential of EATV was assessed in Ehrlich’s tumor-bearing mice. Pathological, histological, and hematological analyses of the EATV-treated animals were performed, and the effect of the treatment on the lifespan was evaluated. The LC-HRMS analysis demonstrated a complex phytochemical profile of EATV comprising more than forty compounds, thirty-six of which were identified. The results showed that the antitumor activity of EATV towards HT-29 cells is due to a pronounced genotoxicity leading to cell cycle arrest and apoptosis of the cells. Pathological studies revealed more massive and frequently detected tumor necrosis, apoptosis, and fistulation in *T. vulgare*-treated mice compared to positive tumor-bearing controls. Furthermore, it was demonstrated that the intraperitoneal application of EATV prolonged the animal’s lifespan.

## 1. Introduction

*Tanacetum vulgare* L. (tansy) is a perennial plant of the family *Compositae* (*Asteraceae*), widely distributed in Europe and Asia, that grows along rivers, screes, and roads, as well as in grassy, weedy places up to 1200 m above sea level. It is considered to be poisonous due to the high content of neurotoxic thujones (isomeric monoterpene ketones) and other toxic substances [1]. However, the main toxic compounds are located in the essential oil [1]. Thus, upon a heat treatment, those substances evaporate, and the aqueous decoctions can be used for medicinal purposes in a relatively large dosage range [2].

Tansy is known as an effective repellent, insecticide, and food preservative. In both folk and modern medicine, it is used as an antiparasitic agent for the treatment of digestive disorders, migraines, fever, ulcers, edema, wounds, etc. [3].

In our previous study [4], we obtained several extracts from the aerial parts (leaves and flowers) of *T. vulgare* of Bulgarian sources, including one by using a biphasic system of ethyl acetate/water (pH 3.0). Our preliminary studies with LC-HRMS (liquid chromatography—high performance mass spectrometry) analyses in negative ionization mode showed that the main compounds of the herb extracts were different caffeoylquinic and dicaffeoylquinic acids and also small quantities of flavonoids and flavonoid-O-glucuronides, such as quercetin and quercetin-O-glucuronide [4]. Various extracts from *T. vulgare* have been shown to possess substantial antitumor activity toward cultured human tumor cells of different origins [4,5,6,7]. Moreover, comparing the extract’s cytotoxicity in cancer and non-cancer cell lines demonstrates selective anticancer effects [4]. The mechanism of cytotoxicity of *T. vulgare* is not yet elucidated, although other species of the same genus (*Tanacetum*) have been shown to block the cell cycle and target apoptosis via the mitochondrial-dependent pathway [8] or throughout a depolymerization of microtubules of the mitotic spindle apparatus [9].

The aim of the present study is to determine the precise composition of the ethyl acetate/water (pH 3.0) fraction from the extract of aerial parts of *T. vulgare* and to obtain data about its antitumor potential in vitro on the human colorectal adenocarcinoma HT-29 cells and in vivo in a mouse model of the solid form of Ehrlich’s breast carcinoma.

## 2. Materials and Methods

### 2.1. Materials

*T. vulgare* samples were supplied by Vemo 99 Ltd. (Sofia, Bulgaria). Dulbecco’s Modified Eagle Medium (DMEM), fetal bovine serum, antibiotic solution (penicillin–streptomycin), phosphate buffered solution (PBS), and trypsin–EDTA solution (2.5 g/L trypsin and 0.2 g/L EDTA) were purchased from Merck (Darmstadt, Germany). Annexin V/PI Apoptosis Detection Kit: sc-4252 AK was the product of Santa Cruz Biotechnology, Inc., Dallas, TX, USA. The other chemicals were purchased from Sigma-Aldrich (Darmstadt, Germany), unless otherwise stated below.

### 2.2. Plant Material and Extraction

Aerial parts (leaves and flowers) of *T. vulgare* from the supplier were in the form of a dried powdered crude ethanol extract. The ethyl acetate/water fraction was obtained as previously described [4]. In brief, 5 g of the powdered material were mixed with 20 mL of water acidified to pH 3.0 with 6 N hydrochloric acid. The extraction was performed twice subsequently with 15 and 10 mL ethyl acetate. The organic phases were combined, dried with sodium sulfate, and the solvent was partially removed in vacuo. Then, diisopropyl ether was added, and the formed precipitate was filtered and dried.

### 2.3. LC-HRMS Analyses

The phytochemical composition of EATV was analyzed using a Q Exactive hybrid quadrupole-Orbitrap mass spectrometer (Thermo Scientific Co., Waltham, MA, USA) equipped with a TurboFlow^®^ LC system and a heated electrospray model HESI II on IonMax^®^ (Thermo Scientific Co., USA). The chromatographic separation of analytes was carried out by Hypersil Gold column (100 mm × 2.1 mm i.d., 1.9 μm) using the following mobile phases: A: 0.1% formic acid in water and B: 0.1% formic acid in acetonitrile at a flow rate of 300 μL/min and gradient: 0% B for 1 min, 30–90% B for 30 min, 90% B for 5 min, 90–0% B for 2 min, and 0% B for 2 min.

Full-scan spectra over the *m*/*z* range 80–1200 were acquired in negative ion mode at resolution settings of 70,000. All MS parameters were optimized for sensitivity to the target analytes using the instrument control software program. Q Exactive parameters were: spray voltage 4.0 kV, sheath gas flow rate 32, auxiliary gas flow rate 10, spare gas flow rate 3, capillary temperature 320 °C, probe heater temperature 300 °C, and S-lens RF level 50. All ion fragmentation (AIF) mode of operation for the mass analyzer was used to identify the extract’s compounds. Optimized values of the collision energy were HCD 25%. Data acquisition and processing were carried out with the Xcalibur software package, version 2.4 (Thermo Scientific Co., USA). Calculations for theoretical *m*/*z* values were made by Mass Frontier 5.1 Software program (Thermo Scientific Co., USA). Extracts of tansy (3 mg) were dissolved in 1 mL of 0.1% formic acid buffer by ultrasound-assisted extraction for 15 min, and 10 μL were injected for LC–HRMS analyses.

### 2.4. Cell Culturing

Human colorectal adenocarcinoma cell line HT-29 (HTB-38) was obtained from the American Type Culture Collection (ATCC; Manassas, VA, USA), and human keratinocyte cell line HaCaT (CVCL_0038) was obtained from the CLS Cell Lines Service (Eppelheim, Germany). Cells were routinely grown in Dulbecco’s modified eagle’s medium—high glucose (DMEM 4.5 g/L glucose), supplemented with 10% fetal bovine serum and antibiotics in usual concentrations in a humidified atmosphere with 5% CO_2_ at 37.5 °C. The cultivation was performed in 25 cm^2^ tissue culture flasks until about 80% confluence. Cells were separated by Trypsin-0.25% EDTA.

### 2.5. Cell Viability Assessment

Cell viability was assessed by neutral red uptake assay. Briefly, cells were plated in a 96-well microtiter plate at a density of 1 × 10^3^ cells/100 µL/well, incubated for 24 h, and then treated with EATV applied at concentrations from 3.9 µg/mL to 500 µg/mL. The standard cytostatic 5-fluorourcil (5-FU) was used as a positive control in the experiment. After 72 h treatment, neutral red medium was applied for 2 h. The cells were then washed and treated with the ethanol/acetic acid solution. The absorption was measured on a TECAN microplate reader (TECAN, Grödig, Austria) at a wavelength of 540 nm.

### 2.6. Cell Cycle FACS-Analysis

Cells were treated with 100 μg/mL EATV for 24 h. As negative controls, non-treated cultured cells were used. The trypsinized cells were centrifuged for 10 min at 1000 rpm. After washing with PBS, they were fixed with ice-cold ethanol while stirring. Cells were stored at −20 °C for 12 h before analysis. Then, cells were treated with RNase A (20 µg/mL) for 30 min and stained with propidium iodide (PI, 20 µg/mL). The analyses were carried out on a flow cytometer Becton Dickinson (San Jose, CA, USA). From each sample, 10,000 events were recorded, and the percentage of cells in different cell cycle phases (G1, S, and G2/M) was determined using FlowJoTM v10.8 software (BD Biosciences, San Jose, CA, USA). Data were presented as mean ± SD of three replicates.

### 2.7. Pro-Apoptotic Activity

Pro-apoptotic activity of EATV was assessed using the Annexin V-FITC/PI apoptosis detection kit (Santa Cruz Biotechnology, Dallas, TX, USA) according to the instructions of the supplier. In brief, HT-29 cells at a density of 1 × 10^5^ cells/well were seeded in a 6-well plate and treated with 100 μg/mL EATV for 24 h. Non-treated cells were used as controls. The cells were trypsinized, centrifuged for 10 min at 1000 rpm, and washed twice with PBS. Then, they were re-suspended in a binding buffer (0.01 M Hepes/NaOH, pH 7.4 containing 0.14 M NaCl and 2.5 mM CaCl_2_). Annexin V-FITC (5 µL) and PI (5 µL) were added to 100 µL of the cell suspension. After incubation for 15 min at room temperature, 10,000 cells were analyzed from each sample with a flow cytometer (BD LSR II) using Diva 6.1.1 Software (BD Biosciences, USA).

### 2.8. Genotoxicity Assay

The assay was performed as described in our previous paper [10]. Cells were treated with 100 μg/mL and 150 μg/mL EATV for 4 and 24 h. Untreated cells from the same line were used as a negative control. Cells treated with 150 mM hydrogen peroxide (H_2_O_2_) for 5 min were the positive control. Cell preparation, electrophoresis conditions, comet staining (silver nitrate), and methods, instrumentation, and statistical data processing are detailed in the above-referenced publication [10]. To determine the DNA percentage in the tail, we used the classification of Noroozi et al. [11]: class 0 (no damages)—1–5%; class 1 (low damages)—5–25%; class 2 (middle damages)—>25–45%; class 3 (high damages)—>45–70%; class 4 (very high damages)—>70%. The index of DNA damages was calculated using the formula proposed by Azqueta et al. [12]:

DNA damage index = 0x(n) + 1x(n) + 2x(n) + 3x(n) + 4x(n)

n = number of cells in each category.

Thus, the count of 100 cells gives the result in the area 0 to 400 units.

### 2.9. In Vivo Experiments

Mature albino mice of 20 g b.w. were purchased from the breeding base for laboratory animals in Slivnitza, Bulgaria and used in the experiment. The experiments were performed in the Institute of Experimental Morphology, Pathology and Anthropology—Bulgarian Academy of Sciences, in accordance with the national regulation No 20/01.11.2012 regarding laboratory animals and animal welfare, the European directive 2010/63/EU of the European Parliament, and of the Council of 22 September 2010 on the protection of animals used for scientific purposes. The study was approved by the Bulgarian Agency for Food Safety, approval number 282, from 24 September 2020. The animals were kept in plastic cages and fed and watered ad libitum. Experimental animals were inoculated with Ehrlich’s ascites carcinoma cells by a single subcutaneous (s.c.) injection of 0.2 mL suspension of 1 × 10^6^ cells/mL into the hind leg to develop a solid form of Ehrlich’s mammary gland carcinoma; five animals inoculated with PBS were used as a negative control. The experiment was started after a palpation of a tumor mass. The animals were randomly divided into three groups: 10 untreated controls and 10 mice treated with EATV by i.p. injections of 0.2 mL with a single dose of 20 mg/kg b.w., and 10 mice treated i.p. with the standard cytostatic 5-FU 15 mg/kg b.w. Both EATV and 5-FU were applied six times at regular time intervals during the period of 18 days. After the treatment was completed, half of the animals were sacrificed, blood was collected for testing, and tissue samples of the tumor mass, liver, pancreas, kidney, and lung were collected. The remaining animals from each group were used for survival tests. The tissue samples were fixed and processed according to the standard procedure, and the sections were stained with H&E (hematoxylin-eosin). Histopathological observations were made under a microscope, Leica DM5000B (Wetzlar, Germany). Blood counts were performed on a Mindray BC 2800 Vet analyzer (Mindray, Shenzhen, China) and included the following hematological parameters: leukocytes (WBCs), erythrocytes (RBCs), hemoglobin (HGB), platelets (PLTs), and differential white blood cell count.

### 2.10. Statistics

The results of all the tests were expressed as mean values ± SD of three replicates for each experiment. Statistical analyses included the Shapiro–Wilks test to confirm normality and one-way analysis of variance (ANOVA) with Bonferroni post hoc test for multiple comparisons (GraphPad Prism 8.0 software) Statistical significance was denoted * *p* < 0.05, ** *p* < 0.01, and *** *p* < 0.001. Nonlinear curve fit analysis was used to determine the IC_50_ concentrations.

## 3. Results and Discussion

### 3.1. LC-HRMS Analyses

**Figure 1:**
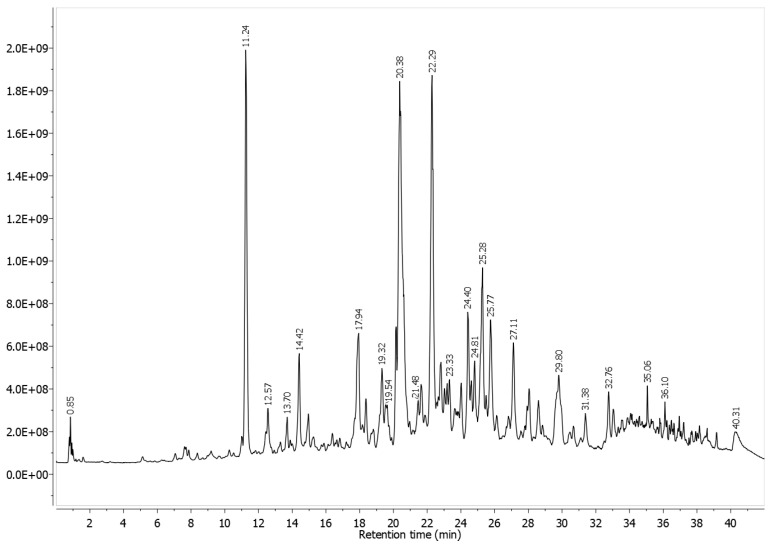
Total ion chromatogram (TIC) of the *T. vulgare* fraction obtained by using biphasic system ethyl acetate/water (pH 3.0).

The nonvolatile compounds derived from EATV were determined by the LC-HRMS method in the negative ionization mode. Compounds were identified via MS and MS/MS data analyses and were compared to the previous data. The total ion chromatogram (TIC) of the EATV is presented in Figure 1. The obtained data were summarized in Table 1. Four groups of substances were identified, the predominant components being hydroxycinnamoyl quinic acids (nine substances) and flavonoids and their derivatives (twenty-three substances). The major nonvolatile compounds were 5-caffeoylquinic acid, 3,5-dicaffeoylquinic acid, and 4,5-dicaffeoylquinic acid. Generally, the major flavonoid was luteolin. Nine unidentified substances were also detected.

### 3.2. In Vitro Anticancer Activity

**Figure 2:**
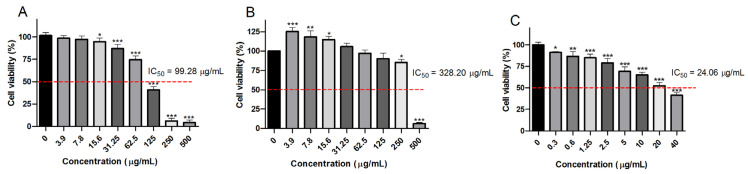
Effect of ethyl acetate/water fraction from *T. vulgare* extract on the viability of HT-29 and HaCaT cells assessed by neutral red uptake assay at 72 h. (**A**) HT-29 cells treated with EATV; (**B**) HaCaT cells treated with EATV; (**C**) HT-29 cells treated with 5-FU (positive control). The data are expressed as the mean ± SD; (*n* = 5); Statistics: one-way ANOVA with Bonferroni post hoc; * *p* < 0.05, ** *p* < 0.01, and *** *p* < 0.001 indicate significant differences compared to the untreated control cells.

*T. vulgare* is commonly used for the treatment of digestive disorders, as it has been found to strengthen the muscles and improve the peristalsis of the gastrointestinal tract and to ameliorate the symptoms of duodenal or stomach ulcers [3]. Nevertheless, the effects of *T. vulgare* extracts on colorectal carcinoma are not elucidated. The in vitro effects of EATV on the cell viability and proliferation of human colorectal adenocarcinoma cells HT-29 were analyzed by the neutral red uptake assay (Figure 2A). Non-tumorigenic human keratinocyte cells line HaCaT was used to assess the selectivity of the EATV cytotoxicity (Figure 2B). The standard cytostatic drug, 5-fluorouracil (5-FU), was used as a positive control (Figure 2C).

The treatment with EATV in a concentration range from 15.6 μg/mL to 500 μg/mL induced a statistically significant and concentration-related decrease in HT-29 cancer cell viability. The lower tested concentrations did not significantly affect the cell viability. The cytotoxic effects of EATV were significantly less pronounced in the normal HaCaT cells, and even a slight increase in cell viability was detected at the low concentrations. A comparative assessment of the effects of EATV and 5-FU demonstrated a higher anticancer effect of the standard antineoplastic agent. Based on the results obtained, the half inhibitory concentration of EATV was determined for both cancer and non-cancer cell lines. The significantly higher cytotoxicity and lower IC_50_ value determined in the colorectal carcinoma cells is an indication of the selective anticancer effect of EATV.

### 3.3. Effect on the Cell Cycle

**Figure 3:**
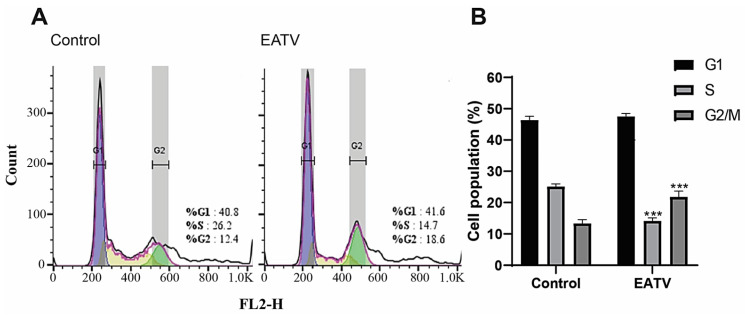
Effect of ethyl acetate/water fraction of *T. vulgare* extract applied for 24 h at a concentration of 100 μg/mL on the cell cycle progression of HT-29 cells. (**A**) Graph of one of the three independent experiments conducted. (**B**) Cell populations in different phases of the cell cycle. The data are expressed as the mean ± SD; (*n* = 3); Statistics: one-way ANOVA with Bonferroni post hoc; *** *p* < 0.001 indicates significant differences compared to the untreated control cells.

The effects of EATV on the cell cycle and its possible pro-apoptotic activity were evaluated using flow cytometry analyses. In order to study the influence of the extract on the cell cycle of HT-29 cultured cells, we used a concentration approximating the determined IC_50_ value (100.0 μg/mL) applied for 24 h (Figure 3).

The results of the flow cytometric analysis demonstrated that the treatment of HT-29 carcinoma cells with EATV induced significant alterations in the cell distribution in the different phases of the cell cycle. From Figure 3, a visible arrest of the G1/S transition in treated cells is seen. Additionally, a statistically significant G2/M arrest was also detected. The G1 phase checkpoint regulates the entrance of normal cells into S phase, in which DNA replication occurs. In many malignant carcinomas, however, the main components of the G1 checkpoint, such as Rb protein, cyclin D1, and p16INK4a, are either altered or differently expressed, leading to an abrogation of the cell cycle control [13]. Similarly, G1/S arrest has been recently reported in MCF-7 (human luminal breast carcinoma) cells induced by a hexane extract from another representative of the *Tanacetum* genus—*Tanacetum polycephalum*, leading to apoptosis through the mitochondrial pathway [8]. The authors have found that the sesquiterpene 8β-hydroxy-4β,15-dihydrozaluzanin C is responsible for the blockage. Since EATV does not contain this sesquiterpene, it can be concluded that mono- and dicaffeoylquinic acids or the accompanying flavonoids can also cause a G1/S transition arrest in HT-29 cells. Additionally, a statistically significant G2/M arrest was seen in Figure 3B. The observed arrest in our experiment of the G1/S transition in treated cells is probably due to a lack of DNA integrity or DNA damage, while G2/M arrest can be explained by the presence of damaged DNA, which must be repaired before mitosis. It has been reported that flavonoids from *Tanacetum gracile* depolymerize the microtubules in MCF-7 cells [9], demonstrating the involvement of the mitosis checkpoint in the cell cycle arrest. Since the representatives of one and the same genus contain similar flavonoids, this might be the case in our experiment, as well.

### 3.4. Pro-Apoptotic Activity

**Figure 4:**
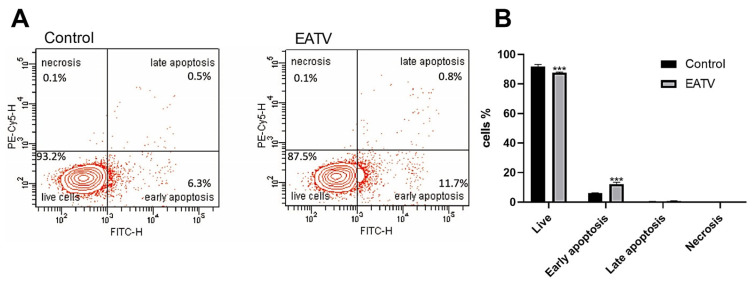
Pro-apoptotic effect of ethyl acetate/water fraction of *T. vulgare* extract on HT-29 cells. (**A**) Graph of one of the three independent experiments conducted. (**B**) Number of live, apoptotic, and necrotic cells after 24 h of the extract application. The data are expressed as the mean ± SD; (*n* = 3); Statistics: one-way ANOVA with Bonferroni post hoc; *** *p* < 0.001 indicates significant differences compared to the untreated control cells.

The pro-apoptotic activity of EATV on HT-29 cells was analyzed using the same concentration (100 μg/mL) for 24 h (Figure 4). The presented results indicate that EATV had a slight pro-apoptotic activity, increasing the number of cells in both early and late apoptosis with a more pronounced effect on early apoptosis. This result shows a similarity with the research of Karimian et al. [8], which demonstrates the pro-apoptotic influence of *Tanacetum polycephalum* (L.) hexane extract on MCF-7 cells. The authors also found a substantial increase in early apoptosis and a much lower increase in late apoptosis. Although neither the extract nor the targeted cells are the same, it can be supposed that the representatives of the genus *Tanacetum* affect the cells by targeting them to apoptosis.

### 3.5. Comet Assay

Chemotherapeutic agents in the treatment of cancer cause significant DNA damage, cell cycle arrest, and apoptosis of the cancer cells. DNA fragmentation of tumor cells is used to select potential new therapeutic agents [14], making it also suitable for evaluating the anticancer potential of our plant extract. The comet assay is a very sensitive and rapid quantitative technique. The assay allows direct measurement of DNA breaks in eukaryotic cells based on the amount of DNA migrating from the cell nucleus during electrophoresis, in the form of a characteristic comet-like pattern. The most widely used version of the method is the alkaline comet assay [15].

**Figure 5:**
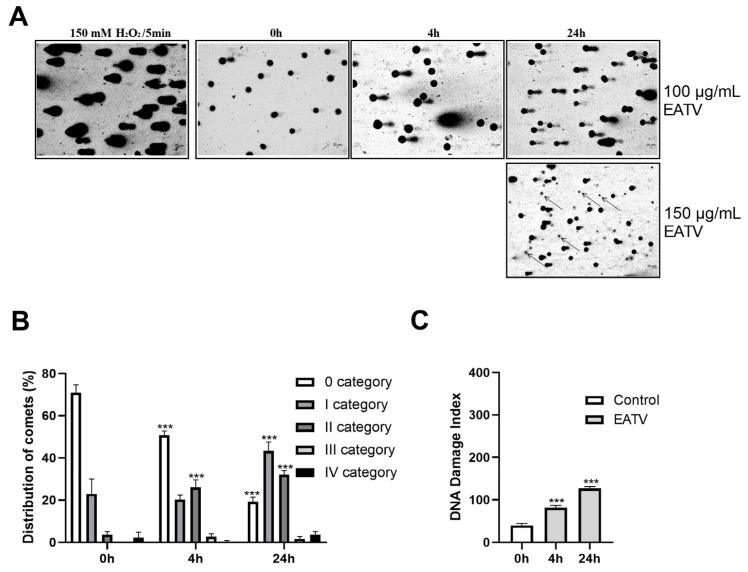
Alkaline comet assay of HT-29 cells treated with ethyl acetate/water fraction of *T. vulgare* extract for 4 and 24 h. (**A**) microphotographs of the cells treated with H_2_O_2_ or EATV; 20× objective; arrows—dead cells, not analyzed by the software; (**B**) cells distribution according to their categories; (**C**) graph of the DNA-damage index. The data are expressed as the mean ± SD (*n* = 3); Statistics: One-way ANOVA with Bonferroni post hoc; *** *p* < 0.001 indicates significant differences compared to the untreated control cells.

The potential genotoxicity of EATV was analyzed by the comet assay in alkaline conditions. The results from the genotoxicity assay are presented in Figure 5. The treatment with H_2_O_2_ (positive control) induced a formation of comets with small heads and very thick and long tails, indicating a high degree of DNA damage. The intensity and length of the comets formed after treatment of the cells with 100 μg/mL EATV increased with the extension of the treatment time. At the high concentration (150 µg/mL) applied for 24 h, a large number of dead cells began to appear.

The presented results demonstrated that the treatment of the cells with 100 µg/mL for 4 h leads to an accumulation of comets mainly of class 2 at the expense of the decrease in comets in class 0 on the 24th hour, a significant increase in the number of comets of class 1 is seen and the increase in those of class 2 continues. No statistically significant increase in the number of cells of classes 3 and 4 was observed (Figure 5B). A gradual increase of the DNA-damage index with the extension of the treatment time was found (Figure 5C).

### 3.6. In Vivo Anticancer Activity

The in vivo experiments were performed on a mouse model of Ehrlich’s tumor. The tumor of Ehrlich is a poorly differentiated, easily transplantable mammary gland carcinoma that resembles the most chemotherapy-sensitive human breast cancers. The mouse tumor-bearing models are widely used to study the effect of potential therapeutics of synthetic or natural origin [16,17] and to assess their potential for application in medicine. A number of in vitro studies have shown a marked antitumor effect of different *T. vulgare* extracts in human tumor cell lines of mammary gland origin [4,8,9]. However, the anticancer potential of this plant species was not confirmed in animal breast cancer models. In a previous study [2], a total aqueous extract from leaves of *T. vulgare* was administered i.p. in healthy mice in order to determine the toxic doses. The authors reported that the lowest-observed adverse effect levels (LOAEL) were 1.5 g/kg b.w. In our study, we administered the Ehrlich carcinoma cells by subcutaneous injections (s.c.) in order to obtain a solid form of the tumor as a model system for assessment of the anticancer efficacy of the experimental therapy with EATV. The EATV was administered in the form of six injections, each containing 20 mg/kg b.w. and a total quantity of 120 mg/kg b.w., a dose much lower than that used in the above-cited acute toxicity study. A standard cytostatic drug, 5-fluorouracil (5-FU), was applied at a single dose of 15 mg/kg b.w. and a total dose of 90 mg/kg b.w. using the same treatment regimen. During the experiment, the animals treated with EATV and 5-FU and the control tumor-bearing mice did not show any substantial differences in their behavior, food and water consumption, and the general condition. Gross pathology also revealed comparable macroscopic observations, such as enlarged spleens (frequent finding in Ehrlich’s tumor-bearing mice), normal pancreas, kidneys, and lungs. However, the liver of *T. vulgare*-treated mice had slightly fragile consistency. In several animals, solid tumors with severe central necrosis with fistulation were observed, more pronounced in the groups treated with the EATV (*n* = 5) and 5-FU (*n* = 6), compared to the control group (*n* = 1).

**Figure 6:**
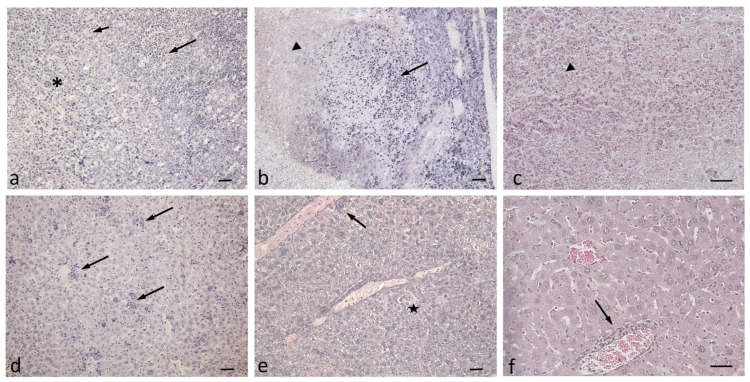
Histopathological examination of solid tumor masses and livers of experimental Ehrlich tumor-bearing animals. (**a**) tumor of a control untreated with the extract: tumor macrophages (asterisk), mitotic figures (short arrow), lymphocyte and neutrophil infiltrations (long arrow); (**b**) tumor mass of an extract-treated mouse: colliquative necrosis (arrowhead), lymphocyte and neutrophil infiltrations (long arrow); (**c**) tumor of a 5-FU-treated mouse: cell debris, necrosis (arrowhead); (**d**) liver of a control mouse: abundant lymphocyte and neutrophil infiltrations (long arrows); (**e**) liver of an EATV-treated mouse: few hepatocytes with hydropic degeneration (star), small number of white blood cells (long arrow); (**f**) liver of an 5-FU-treated mouse: perivascular white blood cell infiltrations (long arrow). Scale bar = 50 µm.

In addition, microscopic studies did not show substantial changes in the pancreas, kidneys, and lungs. The histopathological findings in the tumors and livers of the three groups of animals are presented in Figure 6. In the untreated tumor-bearing controls (Figure 6a), typical tumor cells with pronounced anisocytosis, large and irregular in size nuclei, and prominent nucleoli were observed. Likewise, cell debris, lymphocytes, neutrophil infiltrations, as well as mitotic figures and tumor macrophages, were observed. Similar findings were noticed in the tumor masses of animals treated with EATV and 5-FU, with a difference concerning several animals with massive central lesions of colliquative necrosis of tumor masses and abundant cell debris and more pronounced lymphocyte and neutrophil infiltrations (Figure 6b,c). Mice treated with 5-FU had notably necrotic morphological tumor features of cellular damage and numerous foci with apoptotic cytomorphology, where tumor-associated macrophages were also observed. On the basis of the histopathologic examination, scoring of the observed lesions was performed to detect biologic differences in the non-treated and EATV- and 5-FU-treated groups (Table 2).

The liver architecture of the controls appeared quite normal except for the abundant lymphocyte and neutrophil infiltrations (Figure 6d). In contrast, in the livers of EATV-treated animals (Figure 6e), few focal hydropic degeneration areas of hepatocytes indicative of excess intracellular water and cytoplasm swelling were observed. An insignificant number of lymphocytes and neutrophils were also present. In mice treated with 5-FU, central veins and portal tracts with perivascular lymphoplasmacytic inflammatory cell infiltrate were frequently observed (Figure 6f).

Organ toxicity, due to extract treatment, was not clearly seen in the treated group, except for liver changes in some animals from the *T. vulgare*-treated group, where focal lesions were found, connected with cell vacuolation and swelling, leading to hydropic degeneration, which might progress to subacute liver injury [18]. The observed acute cell swelling and hydropic degeneration could also be related to the metabolic stress and toxin eruption associated with abundant tumor necrosis after *T. vulgare* ethyl acetate/water extract application and provocation of significant inflammation. Nevertheless, these are potentially reversible processes, which are perceived mainly as sub-lethal cell injuries.

To analyze the effects of the treatment with EATV on Ehrlich tumor-bearing mice, hematological analyses were performed (Table 3).

In the tumor controls, the white blood cells were greatly elevated, while in EATV- and 5-FU-treated animals, it was slightly lowered. Differential white blood cell count demonstrated that this result is due to an elevated percentage of granulocytes accompanied by a lowered number of lymphocytes. Thus, the ratio of neutrophils/lymphocytes was increased. A slightly lowered level of HGB in the treated animals was also found. The elevated percentage of granulocytes could be explained with tumor mass central necrosis, observed more distinctly in *T. vulgare*- and 5-FU-treated groups. This result is typical for cancer necrosis, and it was previously announced that this process is a synergistic consequence of metabolic stress leading to oxidative stress-induced cell death and inflammation [19]. In the central necrotic parts, neutrophil infiltrations were seen, explaining the observed granulocytosis [20,21] and the presence of tumor macrophages. Metastatic tumor foci infiltrating the other organs were not observed in treated animals. In control ones, the tumors were infiltrating the nearby subcutaneous adipose tissue, and in the tumor periphery, focal islets of lipocytes were seen. Such contact between breast cancer cells and fatty tissue is a great risk for prompting tumor growth due to the stimulation of the proliferation and invasion by secreting proteases and pro-inflammatory cytokines and by modulating cancer cell metabolism [22].

**Figure 7:**
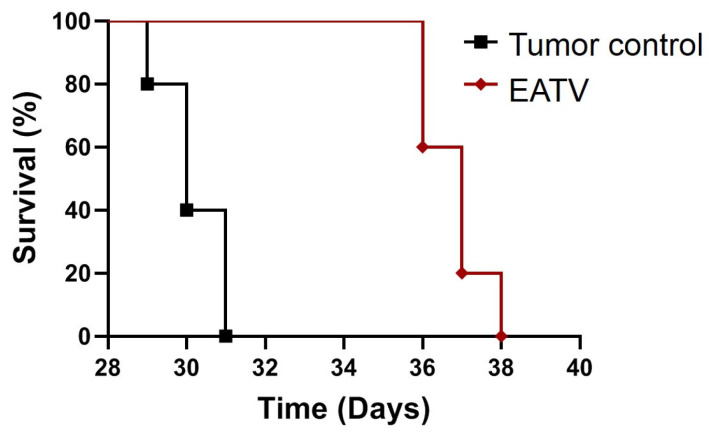
Effects of the treatment with *T. vulgare* ethyl acetate/water extract on survival time of mice with Ehrlich carcinoma.

The anticancer effect of the EATV was also assessed by lifespan monitoring of the tumor-bearing treated and non-treated animals (Figure 7).

As evident from the presented data, positive controls died within 31 days from the beginning of the experiment, while the survival of the treated group was prolonged up to the 38th day. The treatment with EATV extended the mean survival time of the animals from 30.2 ± 0.8 days to 36.8 ± 0.8 days.

## 4. Conclusions

It is shown that the ethyl acetate/water (pH 3.0) extraction of hydrophobic components is more efficient than the traditional use of alcohol–water mixtures. The results of LC-HRMS analysis indicate the presence of thirty-six compounds and nine compounds that were not identified. The main components in EATV are 5-caffeoylquinic acid, 3,5-dicaffeoylquinic acid, and 4,5-dicaffeoylquinic acid. The results also demonstrate that EATV has a pronounced antitumor effect on the human colorectal carcinoma cell line HT-29. It targets the cells to apoptosis due to both a moderate genotoxicity toward the tumor cells and cell cycle arrest at the G1/S and G2/M transitions. The in vivo treatment with the EATV showed anticancer potential in a model of tumor-bearing animals with experimentally provoked Ehrlich’s carcinoma expressed in massive necrotic processes in the tumor tissue, connected with the relevant hematological deviations and the elongation of the animal’s lifespan. To assess *T. vulgare*’s potential for therapeutic applications, further studies are necessary for the identification of the main bioactive compounds and more detailed elucidation of the mechanisms underlying the detected anticancer effects.

## Figures and Tables

**Table 1:** Identification of phytochemical compounds in *T. vulgare* fraction obtained by using the biphasic system ethyl acetate/water (pH 3.0) with LC-HRMS in negative mode.

№	RT (min)	[M-H]^−^ *m*/*z* Error (ppm)	Molecular Formula	MS^2^ Fragments *m*/*z*, (R.I., %)	Proposed Compound
	Benzoic acid derivative				
1	24.40	419.0975 839.2029 ^a^ (−1.59)	C_20_H_20_O_10_	153.0177 (10), 152.0099 (61), 121.0278 (5), 109.0277 (19), 108.0199 (100)	Dihydroxybenzoic acid (O-Benzoyl)glucoside (I)
2	26.13	419.0975 839.2029 ^a^ (−1.59)	C_20_H_20_O_10_	153.0177 (70), 152.0099 (23), 121.0278 (15), 108.0199 (15), 109.0278 (100), 108.0199 (15)	Dihydroxybenzoic acid (O-Benzoyl)glucoside (II)
	Hydroxycinnamoyl quinic acids				
3	7.60	353.0870 (−2.16)	C_16_H_18_O_9_	191.0548 (67), 179.0336 (15), 135.0435 (100)	3-caffeoylquinic acid
4	11.24	353.0870 707.1820 ^a^ (−2.20)	C_16_H_18_O_9_	191.0548 (100)	5-caffeoylquinic acid
5	13.70	337.0925 (−1.23)	C_16_H_18_O_8_	191.0549 (100), 163.0386 (11), 119.0486 (34)	p-Coumaroylquinic acid
6	14.96	367.1024 (−2.90)	C_17_H_20_O_9_	191.0548 (100), 173.0442 (6), 134.0357 (31), 11.0435 (6), 93.0329 (49)	5-Feruloylquinic acid
7	20.16	515.1185 1031.2456 ^a^ (−2.13)	C_25_H_24_O_12_	353.0873 (16), 335.0770 (5), 191.0548 (85), 179.0336 (100), 173.0442 (82), 161.0229 (26), 155.0334 (10), 135.0435 (40)	3,4-dicaffeoylquinic acid
8	20.38	515.1186 1031.2447 ^a^ (−2.64)	C_25_H_24_O_12_	353.0872 (62), 191.0548 (100), 179.0336 (56), 161.0229 (5), 135.0435 (21)	3,5-dicaffeoylquinic acid
9	20.62	515.1183 1031.2448 ^a^ (−2.34)	C_25_H_24_O_12_	353.0872 (23), 191.0548 (100), 179.0336 (22), 161.0229 (5), 135.0435 (8)	1,5-dicaffeoylquinic acid
10	22.29	515.1182 1031.2447 ^a^ (−2.23)	C_25_H_24_O_12_	353.0874 (62), 191.0549 (36), 179.0337 (73), 173.0442 (100), 161.0230 (7), 155.0335 (6), 135.0436 (24)	4,5-dicaffeoylquinic acid
11	23.04	549.1970 (−1.38)	C_27_H_34_O_12_	387.1651 (52), 207.1015 (18), 161.0229 (100)	O-(O-Caffeoyl)glucosyl [(hydroxypentenyl)-3-oxo-cyclopentyl]acetic acid
12	27.93	677.1503 (−1.27)	C_34_H_30_O_15_	515.1187 (27), 353.0873 (87), 335.0768 (18), 191.0548 (66), 179.0336 (80), 173.0441 (80), 161.0229 (100), 135.0435 (44), 111.0435 (5.0), 93.0329 (11), 85.0278 (13)	3,4,5-tricaffeoylquinic acid
	Flavonoids				
13	25.75	285.0397 (−2.10)	C_15_H_10_O_6_	151.0021 (10), 133.0279 (100), 107.0122 (12)	Luteolin
14	25.77	345.0610 (−1.67)	C_17_H_14_O_8_	330.0379 (34), 315.0144 (68), 287.0194 (100), 149.0229 (52)	Quercetagetin 3,6-dimethyl ether
15	26.84	315.0507 (−1.19)	C_16_H_12_O_7_	300.0268 (16), 272.0276 (13), 271.0243 (83), 255.0292 (32), 243.0291 (24), 227.0341 (6), 173.0441 (100), 148.0150 (6)	Nepetin
16	28.60	301.0712 (−1.66)	C_16_H_14_O_6_	286.0473 (8), 285.0399 (15), 214.0628 (5), 213.0547 (6), 201.0183 (12), 199.0388 (15), 173.0441 (16), 164.0099 (54), 161.0228 (81), 151.0020 (55), 136.0149 (79), 135.0435 (39), 134.0356 (51), 125.0227 (10), 108.0199 (100), 107.0121 (47)	Hesperetin
17	28.84	269.0452 (−1.40)	C_15_H_10_O_5_	151.0021 (9), 149.0228 (8), 117.0329 (100), 107.0121 (14), 83.0121 (7)	Apigenin
18	29.59	299.0555 (−1.81)	C_16_H_12_O_6_	285.0358 (11), 284.0321 (66), 256.0370 (100), 255.0292 (16), 227.0341 (23), 211.0389 (7), 199.0388 (7), 183.0438 (5), 151.0020 (36), 125.0227 (5)	Chrysoeriol
19	29.69	315.0506 (−1.35)	C_16_H_12_O_7_	301.0262 (8), 300.0268 (54), 283.0241 (25), 271.0242 (40), 255.0262 (34), 227.0340 (35), 199.0388 (11), 172.0516 (5), 151.0021 (100), 149.9942 (10), 148.0150 (37), 137.0228 (48), 135.0070 (12), 124.0148 (11), 120.0200 (6), 108.2000 (28), 107.0121 (77)	Isorhamnetin
20	29.82	299.0555 (−1.81)	C_16_H_12_O_6_	285.0358 (17), 284.0321 (100), 256.0370 (26), 255.0292 (17), 227.0341 (26), 211.0389 (11), 199.0388 (10), 187.0387 (6), 183.0438 (7), 151.0021 (41), 135.0435 (5), 134.0356 (11), 133.0278 (13), 107.0121 (32), 83.0121 (17)	Diosmetin
21	29.98	329.0662 (−1.38)	C_17_H_14_O_7_	314.0425 (6), 299.01917 (27), 271.0243 (100), 243.0291 (8), 227.0341 (7), 199.0388 (11), 161.0228 (11), 136.9857 (6), 133.0273 (7)	Cirsiliol
22	31.38	359.0766 (−1.87)	C_18_H_16_O_8_	344.0532 (7), 329.0298 (24), 314.0063 (18), 301.0348 (34), 287.0147 (15), 286.0114 (100), 258.0163 (58), 230.0212 (20), 214.0261 (5), 202.0259 (15), 165.9894 (12), 164.9814 (5), 163.0386 (5)	Quercetagetin-3,6,3′(4′)- trimethyl ether
	Flavonoid-O-glucuronides				
23	17.94	463.0875 927.1862 ^a^ (−1.47)	C_21_H_20_O_12_	287.0554 (14), 151.0020 (100), 135.0434 (43), 113.0227 (10)	Eriodictyol-O-glucoronide
24	18.37	477.0668 (−2.15)	C_21_H_18_O_13_	301.0347 (100), 178.9972 (5), 151.0020 (18)	Quercetin-3-O-glucuronide (Miquelianin)
25	19.31	461.0717 (−1.86)	C_21_H_18_O_12_	285.0397 (100), 284.0321(32)	Luteolin-7-O-glucuronide
26	21.66	445.0768 (−1.81)	C_21_H_18_O_11_	269.0449 (100), 175.0234 (7), 113.0227 (22)	Apigenin-7-O-glucuronide
27	22.23	477.1028 (−2.17)	C_22_H_22_O_12_	301.0711 (100), 286.0476 (25), 242.0575 (15), 199.0547 (10), 151.0020 (11), 113.0227 (60)	Homoeriodictyol-O-glucuronide
28	22.80	475.0873 951.1835 ^a^ (−1.81)	C_22_H_20_O_12_	299.0553 (88), 284.0319 (100), 175.0230 (27), 113.0227 (60)	Diosmetin-7-O-glucuronide
29	24.02	505.0982 1011.2038 ^a^ (−1.04)	C_23_H_22_O_13_	329.0661 (76), 314.0428 (58), 299.0191 (100), 271.0243 (5), 175.0234 (18), 168.0049 (25), 161.0228 (25), 151.0022 (7), 137.0227 (21), 125.0227 (10), 113.0229 (52)	Tricin-7-O-glucuronide
30	24.62	519.1135 (−1.70)	C_24_H_24_O_13_	301.0710 (100), 286.0476 (16), 242.05756 (9), 152.0100 (8), 113.0227 (42), 99.0070 (8), 95.0121 (12)	Hesperetin-O-(O-acetyl)glucuronide or Homoeriodictyol-O-(O-acetyl)glucuronide (Isomer I)
31	25.28	519.1133 (−2.18)	C_24_H_24_O_13_	301.0710 (100), 286.0476 (12), 242.05756 (9), 217.0343 (5), 151.0021 (7), 113.0227 (6), 99.0070 (12)	Hesperetin-O-(O-acetyl)glucuronide or Homoeriodictyol-O-(O-acetyl)glucuronide (Isomer II)
	Flavonoid-O-glucosides				
32	18.36	463.0878 927.1828 ^a^ (−0.86)	C_21_H_20_O_12_	301.0328 (31), 300.0269 (100), 285.0398 (13), 284.0322 (5)	Quercetin-3-O-glucoside
33	19.32	447.0925 (−1.73)	C_21_H_20_O_11_	285.0397 (100), 284.0321(32)	Luteolin-7-O-glucoside
34	21.48	505.0982 (−1.04)	C_23_H_22_O_13_	317.1651 (9), 287.0556 (24), 268.0371 (16), 161.0229 (18), 151.0021 (100), 135.0435 (40)	Myricetin-3-O-(O-acetyl)rhamnoside
35	25.14	609.1242 (−1.26)	C_30_H_26_O_14_	447.0926 (5), 323.0767 (7), 285.0398 (100), 284.022(39), 179.0336 (26), 161.0229 (59), 151.0020 (6), 135.0435 (14), 133.0278, 109.0277 (8)	Kaempferol-O-(O-caffeoyl)glucoside or Luteolin-O-(O-caffeoyl)glucoside
	Organic acid				
36	32.76	329.2328 (−1.51)	C_18_H_34_O_5_	229.1436 (23), 211.1332 (100), 209.1171 (14), 183.1377 (40), 171.1012 (89), 165.1270 (11), 139.1112 (43), 137.0956 (10), 127.0762 (37)	9,12,13-trihydroxyoctadec-10-enoic acid
	Unknown compounds				
37	12.45	303.1080 (−1.66)	C_13_H_20_O_8_	201.0393 (27), 101.0591 (100), 87.0070 (71)	Unknown
38	12.57	303.1080 (−1.66)	C_13_H_20_O_8_	201.0393 (43), 101.0591 (100), 87.0070 (89)	Unknown
39	14.42	323.0766 647.1610 ^a^ (−1.89)	C_15_H_16_O_8_	201.0393 (100), 121.0277 (83), 87.0070 (78)	Unknown
40	18.81	281.1391 (−1.59)	C_15_H_22_O_5_	219.1381 (7), 209.0792 (10), 201.1272 (100), 199.1116 (19), 185.0959 (60), 165.0905 (30), 157.0643 (11)	Unknown
41	19.52	281.1391 (−1.59)	C_15_H_22_O_5_	263.1285 (15), 219.1091 (20), 201.1272 (100)	Unidentified Eudesmanolide
42	23.29	399.1289 (−1.98)	C_18_H_24_O_10_	367.1025 (11), 315.0501 (13), 314.0427 (16), 300.0263 (7), 299,0192 (13), 193.0493 (55), 152.0099 (66), 109.0277 (31), 108.0199 (100)	Unknown
43	28.04	503.2491 (−1.44)	C_24_H_40_O_11_	457.2438 (67), 415.2330 (25), 397.2222 (100), 379.2120 (5), 327.1809 (8), 291.1271 (5)	Unknown
44	30.68	531.2503 (−1.40)	C_26_H_44_O_11_	329.2330 (53), 315.1809 (14), 229.1436 (22), 211.1330 (30), 201.0393 (100), 101.0590 (5), 87.0070 (53)	Unknown
45	33.05	329.2328 (−1.51)	C_18_H_34_O_5_	221.1174 (18), 145.0279 (100), 119.0486 (55)	Unknown

^a^ Adduct ion [2M-H]^−^.

**Table 2:** Histopathology scoring of Ehrilch’s tumor-bearing mice treated with ethyl acetate/water fraction of *T. vulgare* extract.

Group	Necrosis	Mitoses	Tumor Macrophages	Lymphocytes and Neutrofils Infiltration	Neovascularisation	Fibrosis
Tumor control	10%	10%	+	++	+	−
EATV	30–50%	5%	+	+++	+	−
5-FU	65–75%	0%	+	++	−	−

(−) none; (+) mild; (++) moderate; (+++) severe.

**Table 3:** Hematological analysis of Ehrilch’s tumor-bearing mice treated with ethyl acetate/water fraction of *T. vulgare* extract.

Treatment	Parameters						
	WBC (10^9^/L)	RBC (10^12^/L)	HGB (g/L)	PLT (10^9^/L)	Differential Count (%)		
					Lym.	Mon.	Gran.
Referent values	0.8–6.8	6.36–9.42	110–143	45–1590	55.8–90.6	1.8–6.0	8.6–38.9
Control	4.5 ± 0.53	8.09 ± 0.37	122.3 ± 6.4	1167 ± 35.5	65.9 ± 4.8	5 ± 0.3	29.1 ± 4.6
Tumor control	**19.4 ± 0.1**	8.64 ± 0.7	117 ±1.03	1060 ± 6.7	50.1 ± 0.7	5.8 ± 0.7	**44.1 ± 0.2**
EATV	**18.2 ±0.3**	7.51 ± 0.5	**98 ± 0.9**	1467 ± 2	**16.7 ± 0.4**	5 ± 0.2	**78.3 ± 0.8**
5-FU	**27.8 ± 2.29**	7.97 ± 1.63	**110 ± 9**	1548 ± 28.7	**18.9 ± 6.2**	3 ± 1.5	**78.1 ± 7.5**

Results are presented as mean ± SD from four independent counts of three animals. Especially high or low counts are marked in bold.

## Data Availability

All data are included in the manuscript.

## References

[1] Radulović N.S., Genčić M.S., Stojanović N.M., Randjelović P.J., Stojanović-Radić Z.Z., Stojiljković N.I. (2017). Toxic essential oils. Part V: Behaviour modulating and toxic properties of thujones and thujone-containing essential oils of *Salvia officinalis* L., *Artemisia absinthium* L., *Thuja occidentalis* L. and *Tanacetum vulgare* L. Food Chem. Toxicol..

[2] Lahlou S., Israili Z.H., Lyoussi B. (2008). Acute and chronic toxicity of a lyophilised aqueous extract of *Tanacetum vulgare* leaves in rodents. J. Ethnopharmacol..

[3] Khatib S., Sobeh M., Faraloni C., Bouissane L. (2023). *Tanacetum* species: Bridging empirical knowledge, phytochemistry, nutritional value, health benefits and clinical evidence. Front. Pharmacol..

[4] Vasileva A.M., Iliev I.A., Lozanov V.S., Dimitrova M.B., Mitev V.I., Ivanov I.P. (2019). In vitro study on the antitumor activity of *Tanacetum vulgare* L. extracts. Bulg. Chem. Commun..

[5] Devrnja N., Anđelković B., Aranđelović S., Radulović S., Soković M., Krstić-Milošević D., Ristić M., Ćalić D. (2017). Comparative studies on the antimicrobial and cytotoxic activities of *Tanacetum vulgare* L. essential oil and methanol extracts. S. Afr. J. Bot..

[6] Ivanescu B., Tuchilus C., Corciova A., Lungu C., Cosmin T.M., Gheldiu A.-M., Laurian V. (2018). Antionidant, antimicrobial and cytotoxic activity of *Tanacetum vulgare*, *Tanacetum corymbosum* and *Tanacetum macrophyllum* extracts. Farmacia.

[7] Babich O., Larina V., Krol O., Ulrikh E., Sukhikh S., Gureev M.A., Prosekov A., Ivanova S. (2023). In vitro study of biological activity of *Tanacetum vulgare* extracts. Pharmaceutics.

[8] Karimian H., Mohan S., Moghadamtousi S.Z., Fadaeinasab M., Razavi M., Arya A., Kamalidehghan B., Ali H.M., Noordin M.I. (2014). *Tanacetum polycephalum* (L.) Schultz-Bip. induces mitochondrial-mediated apoptosis and inhibits migration and invasion in MCF7 cells. Molecules.

[9] Sinha S., Amin H., Nayak D., Bhatnagar M., Kacker P., Chakraborty S., Kitchlu S., Vishwakarma R., Goswami A., Ghosal S. (2015). Assessment of microtubule depolymerization property of flavonoids isolated from *Tanacetum gracile* in breast cancer cells by biochemical and molecular docking approach. Chem.-Biol. Interact..

[10] Sulikovska I., Djeliova V., Kirazov L., Ivanov I., Dimitrova M. (2023). Evaluation of different staining methods and image analyses of the results from a comet assay in human colorectal cancer cells treated with hydrogen peroxide. Acta Morphol. Anthropol..

[11] Noroozi M., Angerson W.J., Lean M.E.J. (1998). Effects of flavonoids and Vitamin C on oxidative DNA damage to human lymphocytes. Am. J. Clin. Nutr..

[12] Azqueta A., Langie S.A.S., Boutet-Robinet E., Duthie S., Ladeira C., Møller P., Collins A.R., Godschalk R.W.L. (2019). DNA repair as a human biomonitoring tool: Comet assay approaches. Mutat. Res. Rev. Mutat. Res..

[13] Niehans G.A., Kratzke R.A., Froberg M.K., Aeppli D.M., Nguyen P.L., Geradts J. (1999). G1 checkpoint protein and p53 abnormalities occur in most invasive transitional cell carcinomas of the urinary bladder. Br. J. Cancer.

[14] Lu Y., Liu Y., Yang C. (2017). Evaluating in vitro DNA damage using comet assay. J. Vis. Exp..

[15] Singh N.P., Mccoy M.T., Tice R.R., Schneider E.L. (1988). A simple technique for quantitation of low levels of DNA damage in individual cells. Exp. Cell Res..

[16] Aldubayan M.A., Elgharabawy R.M., Ahmed A.S., Tousson E. (2019). Antineoplastic activity and curative role of avenanthramides against the growth of Ehrlich solid tumors in mice. Oxidative Med. Cell Longev..

[17] Ali A.D., Badr El-Din K.N., Abou-El-Magd R.F. (2015). Antioxidant and hepatoprotective activities of grape seeds and skin against Ehrlich solid tumor induced oxidative stress in mice. Egypt. J. Basic Appl. Sci..

[18] Abdelhalim M.A.K., Jarrar B.M. (2011). Gold nanoparticles induced cloudy swelling to hydropic degeneration, cytoplasmic hyaline vacuolation, polymorphism, binucleation, karyopyknosis, karyolysis, karyorrhexis and necrosis in the liver. Lipids Health Dis..

[19] Yee P.P., Li W. (2021). Tumor necrosis: A synergistic consequence of metabolic stress and inflammation. Bioessays.

[20] Kitamura H., Kodama F., Odagiri S., Nagahara N., Inoue T., Kanisawa M. (1989). Granulocytosis associated with malignant neoplasms: A clinicopathologic study and demonstration of colony-stimulating activity in tumor extracts. Hum. Pathol..

[21] Hocking W., Goodman J., Golde D. (1983). Granulocytosis associated with tumor cell production of colony-stimulating activity. Blood.

[22] Kolb R., Zhang W. (2020). Obesity and Breast Cancer: A Case of Inflamed Adipose Tissue. Cancers.

